# Phosphate Solubilizing Microorganism *Bacillus* sp. MVY-004 and Its Significance for Biomineral Fertilizers’ Development in Agrobiotechnology

**DOI:** 10.3390/biology11020254

**Published:** 2022-02-07

**Authors:** Raimonda Mažylytė, Justina Kaziūnienė, Liana Orola, Valda Valkovska, Eglė Lastauskienė, Audrius Gegeckas

**Affiliations:** 1 Life Sciences Center, Institute of Biosciences, Vilnius University, LT-10257 Vilnius, Lithuania; egle.lastauskiene@gf.vu.lt (E.L.); gegeckas.audrius@gmail.com (A.G.); 2 Institute of Agriculture, Lithuanian Research Centre for Agriculture and Forestry, LT-58344 Akademija, Lithuania; justina.smel@gmail.com; 3 Faculty of Chemistry, University of Latvia, LV-1004 Riga, Latvia; liana.orola@lu.lv (L.O.); valda.valkovska@lu.lv (V.V.)

**Keywords:** phosphorus, solubilization, organic acids, plant hormones, phosphate-solubilizing bacteria (PSB), biomineral fertilizers

## Abstract

**Simple Summary:**

Agriculture is one of the most significant and important sectors globally. Soil fertility and agricultural production are highly dependent on which plant protection and growth promotion substance is used. Although applying mineral fertilizers can promote rapid crop growth, extensive mineral fertilizer use has reduced soil quality worldwide. Such environmental aspects have led to the search for more sustainable methods to provide plants with nutrients, especially phosphorus. Phosphorus is one of the macronutrients that is indispensable for plant development and growth. Phosphate solubilizing microorganisms are widely applied in agronomic practices to increase the productivity of crops while maintaining the health of soils. Nowadays, special attention is focused globally on ecological farming. One of the new technologies in agriculture is the mixing of agricultural bio-stimulants with mineral fertilizers. Agricultural bio-stimulants are bioproducts that contain substances of natural origin along with beneficial microorganisms. They can be used to activate seeds, plants, and soil. Bio-stimulants are used in plants to improve nutritional efficiency, cause changes in vital and structural processes to affect plant growth, enhance abiotic and biotic stress tolerance and increase the yield and quality of products.

**Abstract:**

In this study, a phosphate solubilizing microorganism was isolated from the soil of an agricultural field in Lithuania. Based on 16S rRNA gene sequence analysis, the strain was identified as *Bacillus* sp. and submitted to the NCBI database, Sector of Applied Bio-catalysis, University Institute of Biotechnology, Vilnius, Lithuania and allocated the accession number KY882273. The *Bacillus* sp. was assigned with the number MVY-004. The culture nutrient medium and growth conditions were optimized: molasses was used as a carbon source; yeast extract powder was used as an organic source; NH_4_H_2_PO_4_ was used as a nitrogen source; the culture growth temperature was 30 ± 0.5 °C; the initial value of pH was 7.0 ± 0.5; the partial pressure of oxygen (pO_2_) was 60 ± 2.0; the mixer revolutions per minute (RPM) were 25–850, and the incubation and the fermentation time was 48–50 h. Analysis using Liquid Chromatography Time-of-Flight Mass Spectrometry (LC-TOF/MS) results showed that *Bacillus* sp. MVY-004 produced organic acids such as citric, succinic, 2-ketogluconic, gluconic, malic, lactic, and oxalic acids. Furthermore, the experiment showed that *Bacillus* sp. MVY-004 can also produce the following phytohormones: indole-3-acetic (IAA), jasmonic (JA), and gibberellic (GA3) acids. In the climate chamber, the experiment was performed using mineral fertilizer (NPS-12:40:10 80 Kg ha^−1^) and mineral fertilizers in combination with *Bacillus* sp. MVY-004 cells (NPS-12:40:10 80 Kg ha^−1^ + *Bacillus* sp. MVY-004) in loamy soil. Analysis was performed in three climate conditions: normal (T = 20 °C; relative humidity 60%); hot and dry (T = 30 °C; relative humidity 30%); hot and humid (T = 30 °C; relative humidity 80%).

## 1. Introduction

Agricultural soil is very rich in insoluble phosphate salt from Ca, Fe, Al, and other elements which plants cannot use for their vegetative process [1]. Phosphorus is essential for plant growth and crop production and is required at an early stage in their growth [2]. Phosphorus mostly accumulates in the young growing parts of the plant where an intensive synthesis of organic matter occurs. The lack of phosphorus in plants results in a decrease in growth rate, productivity, and yield quality [1,3].

Although applying mineral fertilizers can promote rapid crop growth, extensive mineral fertilizer use has reduced soil quality worldwide. The synthesis of mineral phosphorus fertilizers is a highly energy-consuming process and their use has long-term environmental effects related to eutrophication, soil fertility reduction, and carbon release [4]. Such environmental aspects have led to the search for more sustainable means of providing plants with nutrients. Currently, special attention is focused globally on ecological farming. In this context, phosphate solubilizing microorganisms were considered to be the best ecological measure [5,6]. Agricultural bio-stimulants are a bioproduct containing substances of natural origin and beneficial microorganisms which can be used to activate seeds, plants, and soil [7]. This has contributed to great successes in agriculture, including increased availability of insoluble phosphorus and optimized phosphorus uptake [8]. 

The mechanisms of phosphate solubilization by phosphate solubilizing microorganisms involve oxidation-reduction reactions and acidification of organic acids [9]. Organic acids, such as citric, succinic, 2-ketogluconic, gluconic, malic, lactic, and oxalic acids, release H^+^ ions and organic anions into the culture during the oxidation pathway that occurs on the outer face of the cytoplasmic membrane [10]. These organic anions are consumed by supplying metal complex to combine with Ca^2+^ in insoluble Ca_3_(PO_4_)_2_ and H_2_PO_4_^−^/HPO_4_^2−^, released into the culture medium [9]. This microbial metabolism seems to be an effective way to enhance phosphorus availability in agricultural soil [11,12].

Microbial growth dynamics are a key to many applications in biotechnology and ecology [13]. Microorganism cell growth and division can be considered as a complex sequence of chemical reactions [14]. The most fundamental physical and chemical factors affecting the manifestation of microbial growth are the composition of the nutrient medium, temperature, moisture, pH and oxygen levels, and time of cultivation [15]. One of the current research areas for commercial production is the search for optimal conditions for the cultivation of producer microorganisms as biological products [14]. The genus *Bacillus* is one of the most widely distributed in nature and is commonly used for commercial production. The *Bacillus* species has a fast growth rate, a highly adaptable metabolism, and excellent physiological characteristics [16]. However, before upscaling for mass production, cultivation process methods must be improved and optimized in laboratory-scale fermentation [17]. Equally, the development of agricultural preparations requires understanding of the production of microbial metabolites, also known as natural products [18]. Improving methods for the selection of plant growth-promoting microorganisms and determination of their microbial metabolic compounds is important for subsequent application in the agricultural sector [19].

Nowadays, compound fertilizers composted with suitable microbes, organic matter, and low quantities of mineral fertilizers have become the most commonly used method [20]. Biotechnology methods provide an opportunity to apply agronomic biofertilizer microorganisms to the surface of mineral fertilizer granules [21]. A mixture of microorganisms and mineral fertilizers perform several functions simultaneously: protection, fertilizing, and stimulating [19,22]. The use of these fertilizers in agricultural technologies is an additional method to increase plant productivity, and improve soil quality and biological activity. The use of biological mineral fertilizers can reduce the fertilizer dose by 20–30% without losing biological efficiency [23]. This is a new biotechnology which has had great success in ecology, agriculture, regulation of soil functions, and conservation of biodiversity.

Consequently, the aim of this research was to isolate and identify possibly phosphate-solubilizing microorganism from wheat rhizosphere soil, optimize the cultivation and fermentation conditions at laboratory and industrial scales, ascertain the ability to produce organic acids and phytohormones, and determine the potency of microorganisms to increase the efficiency of mineral fertilizers. This is a new biotechnology. The aim was to establish the changes caused by potentially mobile (P_2_O_5_) phosphorus in the loamy soil by the incubation method, after fertilizing the soil with mineral fertilizer (NPS-12:40:10 80 Kg ha^−1^) and spraying this fertilizer with the microorganism *Bacillus* sp. MV-004 (NPS-12:40:10 80 Kg ha^−1^ + *Bacillus* sp. MVY-004). 

## 2. Materials and Methods

### 2.1. Soil Samples Collection

Phosphate solubilizing microorganism was isolated from soil samples from the Panevezys region (55°55’52.7” N 24°16’54.4” E) of Lithuania. The roots of the spring wheat (*T. aestivum*) were extracted from the soil, and the excess friable soil from the plant roots was removed by gently shaking. The rhizosphere soil samples were stored at 4 °C in 150 mL sterile tubes.

### 2.2. Phosphate Solubilizing Microorganism Isolation and Phosphate Solubilizing Activity

One gram (1 g) of each sample was suspended in 100 mL of sterilized distilled water in 500 mL Erlenmeyer flasks. All three flasks were incubated at 30 ± 0.5 °C for 30 min in a shaking incubator at 130 RPM. By the serially diluted method (10^−1^, 10^−2^, 10^−3^, and 10^−4^), soil samples were plated on Pikovskaya’s (PVK) agar medium in triplicate, consisting of 0.5 g yeast extract powder, 10.0 g dextrose, 5.0 g Ca_3_(PO_4_)_2_, 0.5 g (NH_4_)_2_SO_4_, 0.2 g KCl, 0.1 g MgSO_4_ × 7H_2_O, 0.0001 g MnSO_4_ × H_2_O, 0.0001 g FeSO_4_ × 7H_2_O, 20.0 g agar, and dissolved in 1000 mL distilled water; pH was adjusted to 7.0–7.5 before autoclaving at 15 lbs. pressure (121 °C) for 15 min and poured into sterilized Petri dishes [24]. Using the spread plate method, 100 µL of three soil samples were inoculated and plates were incubated at 30 ± 0.5 °C for 5 days [25]. After the incubation period, the colony which had the largest surrounding clear zone was isolated and inoculated again on PVK agar medium. The diameter of the clear zone surrounding the colony indicated phosphate solubilization and was evaluated after 2, 5, 7, 10, and 14 days of incubation and measured as the phosphate solubilization index (**PSI**). **PSI** was calculated as the ratio of the total diameter (colony + clear halo zone) to the colony diameter [26].

 PSI=(Colony diameter+clear halo zone diameter)/(Colony diameter)

### 2.3. Molecular Identification of the Microorganism Strain

A partial sequence of the 16S rRNA genes of the bacterial strain was amplified using the universal primers 27F (5′-GAGTTTGATCCTGGCTCAG-3′) and 1492R (5′ACCTTGTTACGACTT-3′). Sequence analysis of the 16S rRNA gene was completed using the NCBI BLAST tool based on % homology, E-value, and query coverage [27]. The 16S rRNA sequence obtained in this study was deposited in the NCBI GenBank database to obtain the corresponding number [28]. A phylogenetic tree was constructed based on 16S rRNA gene sequences by Neighbor-Joining method, using a distance algorithm with a bootstrap of 1000, with Molecular Evolutionary Genetics Analysis (MEGA X) software [29].

### 2.4. Microorganism and Inoculant Preparation

The microorganism used in this study was incubated at 30 ± 0.5 °C for 24 h in Petri dishes containing LB agar medium consisting of 10.0 g tryptone, 5.0 g yeast extract powder, 10.0 g NaCl, and 20.0 g agar, and dissolved in 1000 mL purified water; pH was adjusted to 7.3–7.7 before autoclaving at 15 lbs. pressure (121 °C) for 15 min [30]. An individual colony was multiplied in an Erlenmeyer flask containing 100 mL of sterile LB liquid medium. Once purified, the isolate was stored at −80 °C in the same LB liquid medium with 20% (*v*/*v*) glycerol.

### 2.5. The Nutrient Medium Composition and Fermentation Process Parameters’ Optimization

Primarily, the phosphate solubilizing microorganism (PSM) was incubated for 48 h at 30 ± 0.5 °C in a shaking incubator at 130 RPM in 2000 mL Erlenmeyer flasks containing 200 mL F004 liquid medium under different nutrients, in this sequence: carbon sources, including glucose, sucrose, starch, glycerol, molasses; organic sources, including yeast extract powder, soybean peptone, agro-peptone, meat extract powder, and bull extract powder; and nitrogen sources, including KNO_3_, CH_4_N_2_O, (NH_4_)H_2_PO_4_, (NH_4_)_2_SO_4_, NH_4_Cl. When the composition of the culture medium was determined, experiments were then executed on cell growth parameters: first, temperature at 20–40 °C with 5 °C increments; then pH value 6.0–8.0 with 0.5 increments. The optimization was performed three times independently. Analysis was conducted by VR-2000 Spectrophotometer by determining the optical density at 600 nm (OD_600_) and using the serial dilution method by counting CFU mL^−1^ [31,32].

### 2.6. Fed-Batch Fermentation Process Optimization and Bacterial Broth Samples Collection

Fed-batch fermentations were performed using the 5 L laboratory-scale bioreactor (EDF 5.4_1). The fermentation process parameters and the composition of the nutrient medium were chosen according to the experiments that explained the best conditions for the growth of these microorganism cells.

After sterilization at 125 °C for 60 min, the bioreactor vessel was placed on the control block and all connectors were connected correctly. All controlled fermentation parameters were determined: stirrer speed per minute (RPM), partial pressure of oxygen (pO_2_), airflow, temperature, and pH values. Three sterile systems were used, with silicone tubes containing alkaline solution (2M NaOH), acidic solution (2M H_2_SO_4_), and antifoam (silicone-based) solution. One of the same systems (for culture medium and inoculum) was connected to the indicated pumps to enter the fermenter vessel. At the start of the fermentation process, the water supply was opened to maintain the necessary temperature. Alkaline and acidic solutions were used to retain the pH required during the fermentation process. The pO_2_ sensor was calibrated at one point based on airflow and RPM at the maximum value, lifted manually. If foam occurred during the calibration process, an antifoam solution was used to destroy it. In a laminar flow cabinet, under sterile conditions, the inoculum was poured into a sterile system and dosed into the fermenter vessel in which a sterile culture medium was already prepared. Growth parameters of the fermentation process, alkaline, acidic, and antifoam solutions pumps were set in automatic mode. 

Fed-batch fermentation in the 5 L lab-scale bioreactor (EDF 5.4_1) was repeated in triplicate and then the fermentation process for the microorganism cells was optimized at the industrial level with a production line using the 50 L, 500 L, and 5000 L volume bioreactors. Two small-scale bioreactors (50 L and 500 L) were used to grow the required amount of inoculum (10%) for the 5000 L final industrial fermentation.

In the whole fermentation process, samples of the bacterial suspension were collected every two hours to determine glucose concentration variation in the nutrient medium. Before the start of the fermentation process, a culture medium without cell culture was used as a control sample. When the minimum glucose concentration limit was reached during the fermentation, the cells were fed by dosing a new fresh nutrient medium to the bioreactor. Glucose concentration throughout the fermentation was determined using the YSI 2900 Biochemistry Analyzer [33]. Cell feeding was performed with increasing volumes of liquid nutrient medium every 30 min during the fermentation process. The end of the fermentation process was established by microscopic analysis and fermentation process parameter trends. The fermented product was collected in 500 mL sterile bottles every 10, 20, 30, 40, and 48–50 h of the fermentation process. All bottles with samples were stored at 4 °C temperature.

### 2.7. Organic Acids Analysis by Liquid Chromatography Time-of-Flight Mass Spectrometry (LC-TOF/MS)

Fermentation samples were centrifuged at 4200 RPM for 10 min. The resulting supernatant was diluted with acetonitrile (ratio 1:1). The lower fraction was collected and diluted at a ratio of 1:3 with water. The solution was filtered through 0.45 μm filters and analysed by HPLC-MS. The concentration of organic acids was determined by an Agilent 6230 TOF LC/MS system (Agilent Technologies, Waldbronn, Germany) with electrospray ionization (ESI) [34]. The chromatographic separation was carried out on Phenomenex Rezex ROA-Organic Acid H^+^ (8%) column (150 × 4.6 mm) that was incubated at 55 °C. Isocratic elution of 0.5% (*v*/*v*) aqueous formic acid at a flow rate of 0.30 mL min^−1^ was used. The injection volume was 2 μL. ESI-MS was performed in negative ionization mode with settings as follows: fragmentor voltage, 75 V; drying gas flow, 10.0 L min^−1^; drying gas temperature, 285 °C; capillary voltage, 3500 V; nebulizer pressure, 40 psi. The full scan mass range was 50–1100 *m*/*z*. Internal reference masses 112.9856 *m*/*z* and 1033.9881 *m*/*z* were used. The measurements were carried out in triplicate. All data were processed using MassHunter 7.00 software (Agilent Technologies, Waldbronn, Germany). The concentration of organic acids was calculated from standard curves.

### 2.8. Phytohormones Analysis by Liquid Chromatography Time-of-Flight Mass Spectrometry (LC-TOF/MS)

The method of sample preparation was adapted from Castillo et al. [35]. Fermentation samples were centrifuged at 4200 RPM for 10 min. The supernatant aliquot of 3 mL was adjusted to pH 2–3 with 3M HCl. Then 3 mL of ethyl acetate was added. The resulting mixture was extracted by shaking for 5 min followed by centrifugation at 4200 RPM for 5 min. The extraction procedure was repeated three times. The fractions of ethyl acetate were combined and evaporated to dryness with nitrogen gas at 40 °C. The residue was dissolved in 1.0 mL of 20% methanol in water containing 0.1% formic acid, filtered through a 0.45 µm filter and analysed by HPLC-MS. Identification and quantification of indole-3-acetic acid, jasmonic acid, and gibberellic acid were carried out using an Agilent 6230 TOF LC/MS system (Agilent Technologies, Waldbronn, Germany) with electrospray ionization (ESI). The chromatographic separation of phytohormones was performed at 40 °C using a Kinetex C18, 3.0 × 100 mm, 2.6 μm column. The mobile phase consisted of 0.1% aqueous formic acid (A) and 0.1% formic acid in methanol (B). The flow rate was 0.2 mL min^−1^ and gradient elution was performed according to the following program: 0 min, 10% B; 2 min, 10% B; 3 min, 30% B; 4.5 min, 30% B; 5.5 min, 50% B; 8.5 min, 50% B; 14.5 min, 70% B; 16 min, 98% B; 18 min, 98% B; 19 min, 10% B; 23 min, 10% B. The injection volume was 20 μL. ESI-MS was performed in positive ionization mode. The mass spectrometer operating conditions were as follows: drying gas temperature, 320 °C; drying gas flow, 12 L min^−1^; nebulizer pressure, 40 psi; capillary voltage, 3500 V; fragmentor voltage, 130 V. The full scan mass range was set at 50–1000 *m*/*z*. Internal reference masses 121.0509 *m*/*z* and 922.0098 *m*/*z* were used. Each sample was analysed in triplicate. Data processing was carried out using MassHunter 7.00 software (Agilent Technologies, Waldbronn, Germany). The concentration of indole-3-acetic acid, jasmonic acid, and gibberellic acid was determined from standard curves.

### 2.9. Mobile Phosphorus (P_2_O_5_) Concentration Changes in Loamy Soil Using Mineral Fertilizers (NPS-12:40:10) and Biomineral Fertilizers (NPS-12:40:10 + Bacillus sp. MVY-004)

The interaction of fertilizers with soil was studied by the incubation method using a climate chamber and selected loam soil (a fertile soil of clay and sand) from central Lithuania (mobile phosphorus about 260 mg Kg^−1^). Considering the climate in recent years, three test treatments were developed:

1. Normal (T = 20 °C; relative humidity 60%),
2. Hot and dry (T = 30 °C; relative humidity 30%),
3. Hot and humid (T = 30 °C; relative humidity 80%).

The rate of application of fertilizers to the soil was P_2_O_5_ 80 Kg ha^−1^. During the experiments, 4 units of jars were removed from the climate chamber after 2, 4, 6, 8, 10, 12, 14, 16, 18, 20, 22, 24, 26, 28, and 30 days from the start of the experiment.

### 2.10. Bacillus sp. MVY-004 Cells Viability and Activity in Biomineral Fertilizers 

The mineral fertilizers (NPS-12:40:10) and biomineral fertilizers (NPS-12:40:10 + *Bacillus* sp. MVY-004) pellets were made by a granulation process with some manufacturer modifications [36]. Particularly important was studying the viability of the microorganism cells in the new biomineral fertilizers’ biotechnology compositions. In this study, biomineral fertilizer NPS-12:40:10 80 Kg ha^−1^ + *Bacillus* sp. MVY-004 were stored at room temperature (about 24 °C) for 1000 days. *Bacillus* sp. MVY-004 cells’ viability and activity were determined by using the serial dilution method by counting CFU mL^−1^. A two grams (2 g) sample of fertilizer pellets were dissolved in 100 mL of sterile 0.9% saline and the serial dilution method was performed. The required amount of 100 µL of the solution was transferred into Petri dishes with LB agar medium. The plates were incubated at 30 ± 0.5 °C for 24 h. The analysis was performed in triplicate every 1, 8, 20, 26, and 35 months.

### 2.11. Statistical Analysis

All the data were processed with SPSS 13.0 statistical software and are presented as the mean ± standard deviation (SD). One-way ANOVA was used to calculate the data variance, and *p* < 0.05 represents a significant difference.

## 3. Results and Discussion

### 3.1. Isolation, Screening, and Identification of Phosphate Solubilizing Bacteria

Eight phenotypically different bacterial colonies were obtained from three different soil samples, which were collected from Panevezys, Lithuania. Only one bacterial strain showed maximum phosphate solubilization activity as indicated by transparent zone formation on PVK agar plates. The higher zone of PSI was 2.83 in PVK agar media (Table 1).

**Figure 1:**
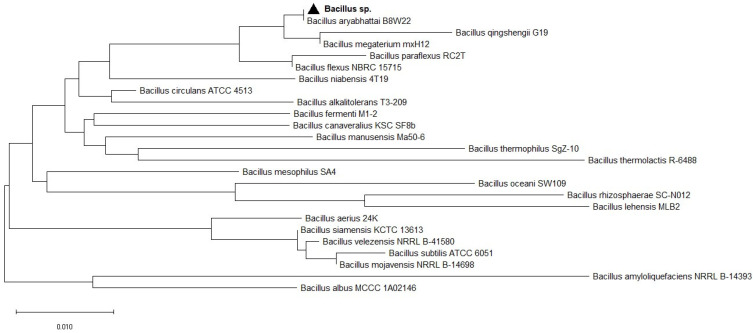
The evolutionary history was inferred using the Neighbor-Joining method. The optimal tree with sum of branch length = 0.50672131 is shown. The percentage of replicate trees in which the associated taxa clustered together in the bootstrap test (1000 replicates) is shown next to the branches. The tree is drawn to scale, with branch lengths in the same units as those of the evolutionary distances used to infer the phylogenetic tree. The evolutionary distances were computed using the Maximum Composite Likelihood method and are in units of the number of base substitutions per site. This analysis involved 25 nucleotide sequences. Codon positions included were 1st + 2nd + 3rd + Noncoding. All ambiguous positions were removed for each sequence pair (pairwise deletion option). There were a total of 1374 positions in the final dataset. Evolutionary analyses were conducted in MEGA X.

Isolate from soil formed colonies that were larger, yellowish colored, opaque, and glossy. Microscopy of the selected bacterial isolate showed that cells were motile, rod-shaped, spore-forming, and gram-negative. Phosphorus solubilizing bacteria were identified based on PCR amplification of the 16S rRNA gene. The 16S rRNA gene sequencing of strain showed 100% sequence homology with *Bacillus aryabhattai* B8W22. The phylogenetic tree was inferred using the Neighbor-Joining method. Evolutionary analyses were conducted in MEGA X: Molecular Evolutionary Genetics Analysis across computing platforms (Figure 1). PSB microorganism was submitted in the NCBI database to Sector of Applied Bio-catalysis, University Institute of Biotechnology, Vilnius, Lithuania and allocated with accession number KY882273. The *Bacillus* sp. Was assigned with the number MVY-004.

### 3.2. Optimization of the Culture Medium and Growth Conditions of the Bacillus sp. MVY-004

**Figure 2:**
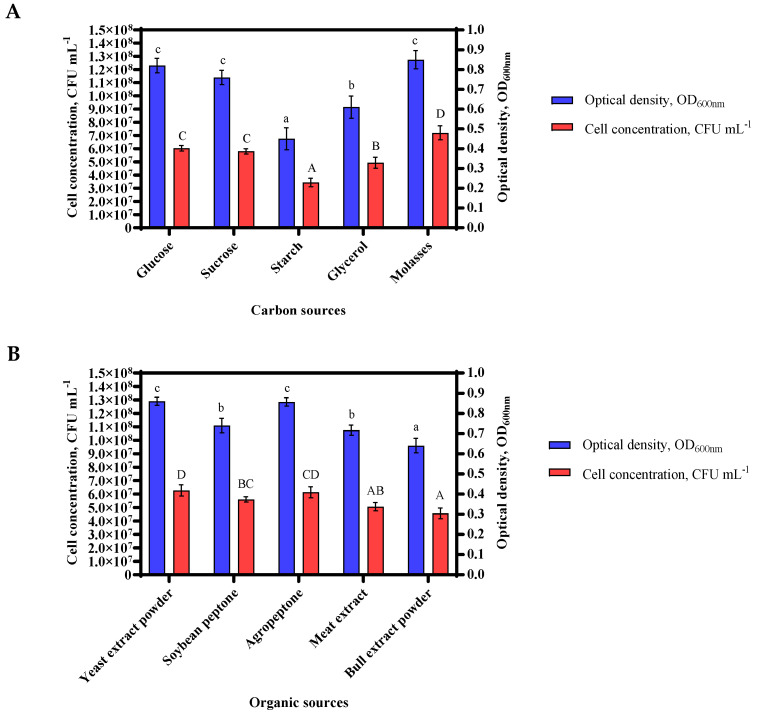

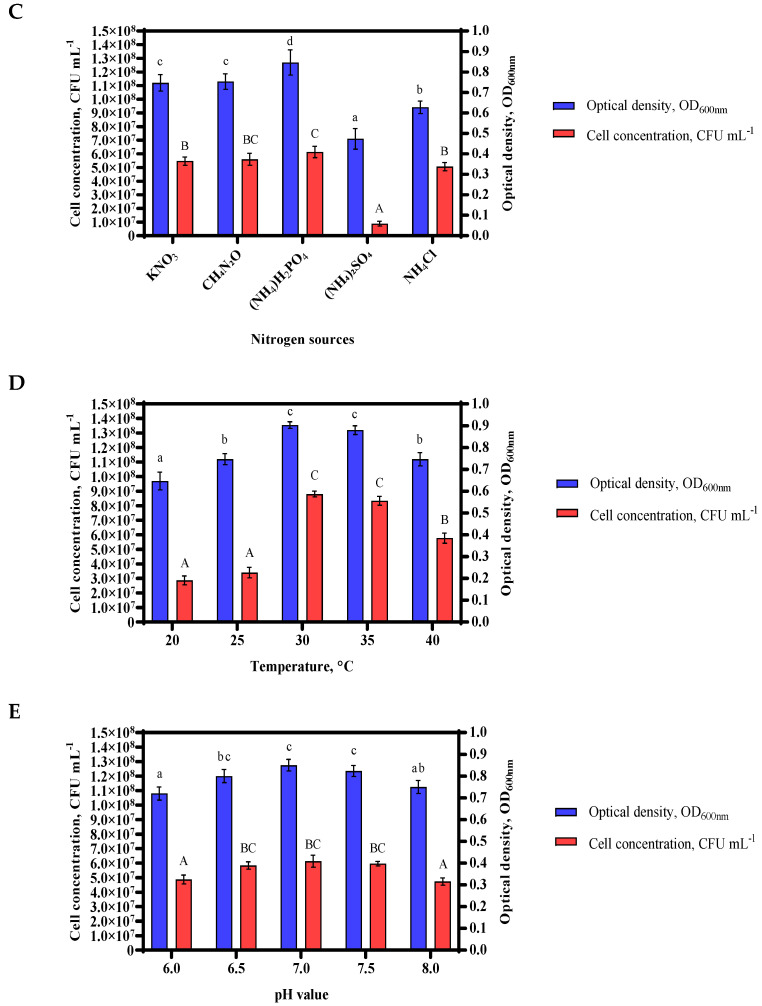
The most suitable source of carbon in the culture medium was molasses (**A**); the organic source in the culture medium was yeast extract (**B**); the nitrogen-containing material selected was ammonium dihydrogen phosphate (**C**). Of the growth parameters, the best temperature value was found to be 30 °C (**D**) and the pH value was maintained at 7.0 ± 0.5 (**E**). Error bar, mean (n = 4) ± standard deviation. Different letters above bars indicate significant differences (*p* < 0.05) in one-way ANOVA.

Glucose, sucrose, starch, glycerol, and molasses was used as the carbon source, the growth efficiency of *Bacillus* sp. MVY-004 was determined as 0.81, 0.76, 0.45, 0.61 and 0.92 at OD_600nm_, respectively (the CFU mL^−1^ was 5.8 × 10^7^, 6.0 × 10^7^, 3.4 × 10^7^, 4.9 × 10^7^ and 9.5 × 10^7^, respectively). This result demonstrated that molasses served as the best carbon source for assisting the growth capacity of *Bacillus* sp. MVY-004 cells. It was also noticed that starch had the lowest cell increment and the value of pH was least different from the control sample, suggesting that starch cannot be utilized as a carbon source by *Bacillus* sp. MVY-004 (Figure 2A).

Next, the effect of organic sources was determined, including yeast extract powder, soybean peptone, agro-peptone, meat extract powder, and bull extract powder of *Bacillus* sp. MVY-004 was 0.86, 0.74, 0.86, 0.72 and 0.64 at OD_600nm_, respectively (the CFU mL^−1^ was 6.3 × 10^7^, 5.6 × 10^7^, 6.1 × 10^7^, 5.1 × 10^7^ and 4.6 × 10^7^, respectively). These results showed a slight difference between the yeast extract and the agro-peptone, but the yeast extract was chosen as an organic source for further study due to the better solubility of the substance (Figure 2B).

The value of growth conditions with various nitrogen sources, including KNO_3_, CH₄N₂O, (NH_4_)H_2_PO_4_, (NH₄)₂SO₄, and NH_4_Cl of *Bacillus* sp. MVY-004 was 0.75, 0.75, 0.84, 0.47 and 0.63 at OD_600nm_, respectively (the CFU mL^−1^ was 5.5 × 10^7^, 5.6 × 10^7^, 6.1 × 10^7^, 8.8 × 10^6^ and 5.1 × 10^7^, respectively). The obtained results showed that the best growth and cell division occurs with NH_4_H_2_PO_4_. On the contrary, using (NH_4_)_2_SO_4_ as a nitrogen source of *Bacillus* sp. MVY-004, the growth of cells was not detected and many bacteria debris were seen microscopically in the bacterial medium (Figure 2C).

The effect of temperature on the growth rate of *Bacillus* sp. MVY-004 at 20, 25, 30, 35, and 40 °C was 0.65, 0.75, 0.90, 0.88 and 0.75 at OD_600nm_, respectively (the CFU mL^−1^ was 2.9 × 10^7^, 3.4 × 10^7^, 8.8 × 10^7^, 8.3 × 10^7^ and 5.8 × 10^7^, respectively). After the incubation period, this test showed that cell adaptation and growth at lower temperatures were slower, and optimal growth temperature was 30 ± 0.5 °C (Figure 2D).

To evaluate the influence of pH on the growth efficiency of *Bacillus* sp. MVY-004, the initial pH value of the nutrition medium was set as 6.0, 6.5, 7.0, 7.5, and 8.0. The growth rate of *Bacillus* sp. MVY-004 in this media was 0.72, 0.80, 0.85, 0.82 and 0.75 at OD_600nm_, respectively (the CFU mL^−1^ was 4.9 × 10^7^, 5.8 × 10^7^, 6.1 × 10^7^, 5.9 × 10^7^ and 4.7 × 10^7^, respectively). After the incubation period with these different pH values, we found that the change in microorganism growth was minimal. Therefore, the most appropriate value of pH was set at 7.0 ± 0.5 (Figure 2E).

### 3.3. Optimization of the Industrial Fermentation Process of the Bacillus sp. MVY-004

**Figure 3:**
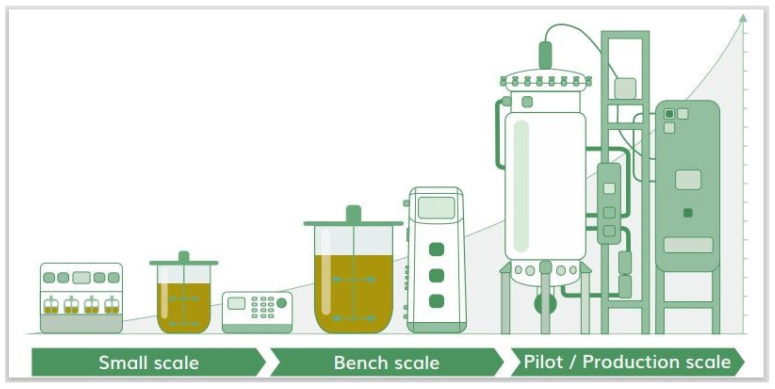

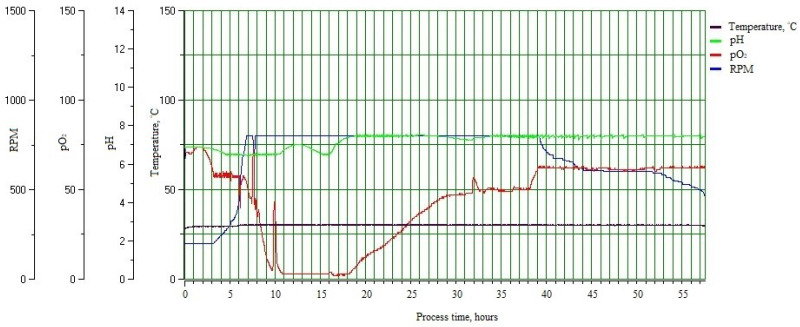
*Bacillus* sp. MVY-004 fermentation process trends at industry-scale (5000 L) bioreactor. Among the trends’ recorded values are: temperature, pH, partial pressure of oxygen, and stirrer revolutions per minute.

After clarification of the best components of the microorganism nutrient medium and growth conditions, the fermentations were performed in the 5 L lab-scale bioreactor (EDF 5.4_1) (inoculum concentration was 1.9 × 10^7^ CFU mL^−1^). At the beginning of the fermentation (1–4 h), the Lag phase of bacterial biomass growth occurred [37]. In this phase, the cells of the microorganism adapted to the new growth conditions when they entered the new environment. From the fifth hour of fermentation, an exponential growth phase began [38]. During this phase, the cells of the microorganism were divided. When the exponential phase changed to the stationary phase of cell growth and glucose concentration in the bacterial broth reached the minimum point, the dosing of the feeding solution began (10 h of the fermentation process) [39]. Cell feeding was performed by dosing the solution at a constant rate of 8–10 mL min^−1^. The feeding was completed at 12 h of the fermentation process. The stationary phase after feeding was up to 16–18 h of the fermentation process. Since the growth had gradually stopped, the phase of cell death began. The fastest change in carbon source concentration was recorded in 10–20 h of the fermentation process. The most intense phase of cell death began at 33 h of the fermentation process. Each fermentation in the 5 L lab-scale bioreactor was stopped after 48–50 h when the carbon source in bacterial suspension was no longer fixed and spores were formed in all cells inside. When all growth conditions were discovered at a laboratory scale, the fermentation process was transferred to the industrial line. The inoculum in the 50 L bioreactor was grown in the 5 L lab-scale bioreactor and transposed to a sterile system connected to the 50 L bioreactor. The fermentation process in this bioreactor went past 15–20 h, when the cells reached a stationary growth phase. When the microorganism growth parameters (RPM and airflow) were reached at the maximum value, the bacterial culture from the 50 L bioreactor was inoculated as a 10% inoculum into the 500 L industrial bioreactor containing the sterile and fresh nutrient medium. Process in the 500 L bioreactor lasted for 4–5 h. When the growth parameters achieved a maximum, 10% volume of the bacterial suspension from the 500 L bioreactor was dosed into the 5000 L bioreactor with the prepared new nutrient medium by production pipeline assistance. These fermentation samples at 48 h were collected and used for the detection of organic acids and plant hormones (Figure 3).

### 3.4. Organic Acids and Plant Hormone Detection in Bacterial Supernatant Using Liquid Chromatography Time-of-Flight Mass Spectrometry (LC-TOF/MS) with External Calibration

Organic acids and plant hormones were determined in the culture supernatant of *Bacillus* sp. MVY-004 at 48 h of the fermentation process in F004 medium using Liquid Chromatography Time-of-Flight Mass Spectrometry (LC-TOF/MS). Seven different organic acids, including citric, succinic, 2-ketogluconic, gluconic, malic, lactic, and oxalic acids were detected. Organic acids were confirmed by comparing LC-TOF/MS results of three pure organic acids as standards. Sterile F004 medium was used as a control sample. Of the seven different organic acids, lactic acid was detected in the largest quantity (419.0 µg mL^−1^), followed by gluconic acid (287.0 µg mL^−1^), succinic acid (257.0 µg mL^−1^), malic acid (232.0 µg mL^−1^), 2-ketogluconic acid (68.0 µg mL^−1^), oxalic acid (34.0 µg mL^−1^) and citric acid (13.5 µg mL^−1^) (Table 2).

LC-TOF/MS was also used to analyze phytohormones. Three different plant hormones, including indole-3-acetic, jasmonic and gibberellic acids, were detected in the bacterial supernatant of *Bacillus* sp. MVY-004. Sterile F004 medium was used as a control sample. Of the three different plant hormones, indole-3-acetic acid was identified in the largest quantity (1.370 µg mL^−1^), following gibberellic acid (0.800 µg mL^−1^) and jasmonic acid (0.173 µg mL^−1^) (Table 3).

The results of the current study have approved the applicability of *Bacillus* sp. MVY-004 isolate to dissolve insoluble inorganic phosphate compounds in the soil. Plant hormones synthesized by this microorganism increase plant root mass, growth and disease resistance, and provide other positive effects.

### 3.5. Variation of Mobile Phosphorus (P_2_O_5_) Concentration in Loamy Soil Using Fertilizers NPS-12:40:10 and NPS-12:40:10 + Bacillus sp. MVY-004

**Figure 4:**
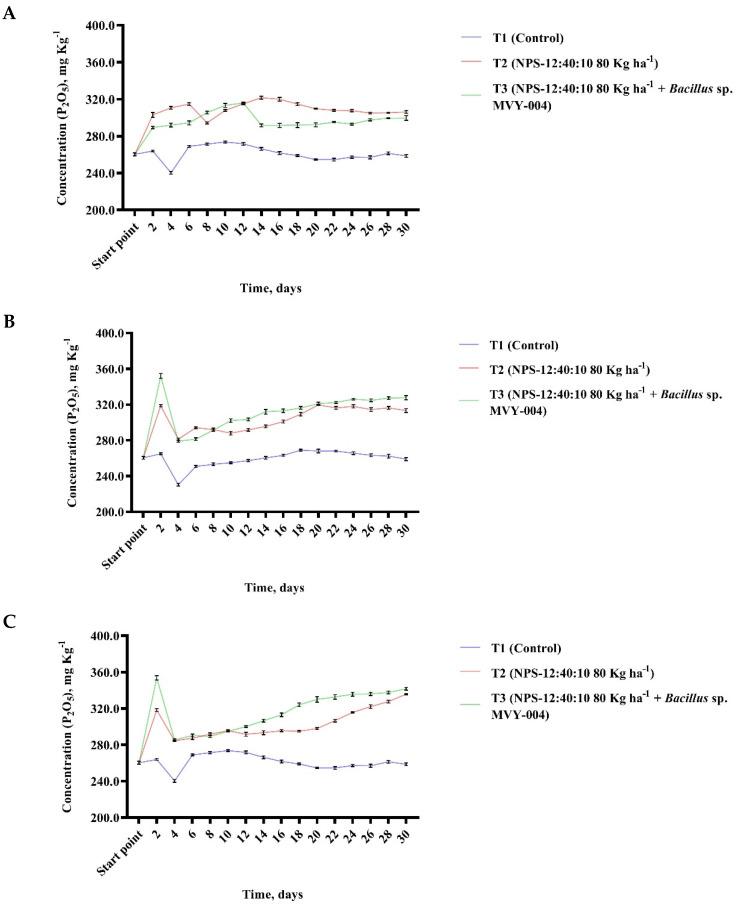

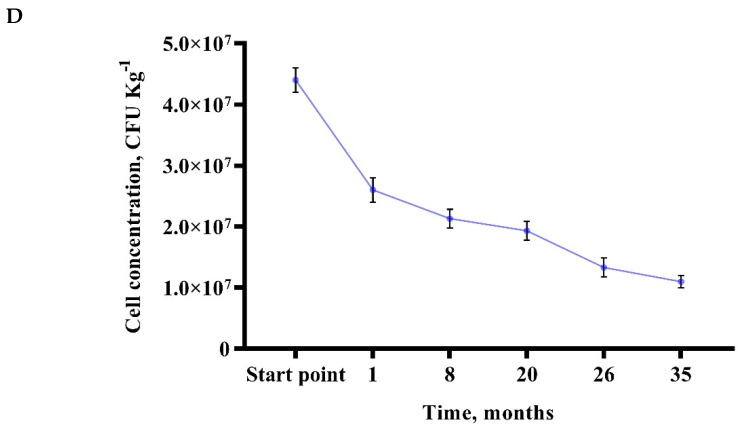
The relationship between phosphorus (P_2_O_5_) concentration (mg Kg^−1^) variations in loam soil in the incubation test with normal (T = 20 °C; relative humidity 60%) conditions (**A**), hot and dry (T = 30 °C; relative humidity 30%) conditions (**B**), hot and humid (T = 30 °C; relative humidity 80%) conditions (**C**) and immobilized *Bacillus* sp. MVY-004 microorganism cells display possible vitality in mineral fertilizers for at least 1000 days (**D**).

The purpose of the research was to scientifically substantiate the effectiveness of using biomineral fertilizers to reduce the loss of nutrients. The experiment was started by selecting normal climate conditions (T = 20 °C; relative humidity 60%) to find out how fast and efficiently the concentration of mobile phosphorus in the loam soil increases using mineral fertilizers (variant T2 (NPS-12:40:10 80 Kg ha^−1^)) and mineral fertilizers mixed with the microorganism *Bacillus* sp. MV-004 (variant T3 (NPS-12:40:10 80 Kg ha^−1^ + *Bacillus* sp. MVY-004)). In analysis under normal condition, it was observed that variant T3 (NPS-12:40:10 80 Kg ha^−1^ + *Bacillus* sp. MVY-004) reaches the highest concentration of mobile phosphorus on day 6 from the beginning of the climate chambers experiment. In the case of variant T2 (NPS-12:40:10 80 Kg ha^−1^), the maximum concentration of mobile phosphorus was found on day 12 from the beginning of the experiment (Figure 4A). Under normal conditions, the concentration of mobile phosphorus in the loamy soil did not determine a significant effect difference between variant T2 (NPS-12:40:10 80 Kg ha^−1^) and T3 (NPS-12:40:10 80 Kg ha^−1^ + *Bacillus* sp. MVY-004).

Based on a scientific article, it was decided to perform experiments in two other variants [40]. Was selected the optimal hot air temperature 30 °C with different relative humidities: 30% and 80%. In hot and dry climate conditions, it was observed that the most effective concentration of mobile phosphorus in the loam soil was observed on day 2, using mineral fertilizers mixed with the microorganism *Bacillus* sp. MVY-004 and concentration decreased suddenly on day 4. In a 30-day experiment, it was observed that in hot and dry conditions the concentration of mobile phosphorus using mineral fertilizers with *Bacillus* sp. MVY-004 microorganism cells achieved better results than using only mineral fertilizers NPS-12:40:10 (Figure 4B).

In hot and humid climate conditions the maximum concentration of mobile phosphorus in the loam soil was detected using mineral fertilizers in combination with *Bacillus* sp. MVY-004 on day 2 and a sudden decrease in concentration was also observed on day 4. On the following days, slight difference in the concentration of mobile phosphorus in the loam soil was also observed when mineral fertilizers and biomineral fertilizers were used in combination with *Bacillus* sp. MVY-004 (Figure 4C).

Experiments with three variants showed that mineral fertilizers together with the microorganism *Bacillus* sp. MVY-004 cells have a rapid effect on the soil. Practically 2–3 days after fertilization the maximum concentration of mobile phosphorus in the soil is reached, which was approximately 10–15% higher than when using only mineral fertilizers NPS-12:40:10. The incubation tests with fertilizers allow the evaluation of the effectiveness of fertilizer over 1–2 months for soil, and with selected climate conditions. It is appropriate to test and evaluate the efficiency of new fertilizers via this method. The experiment should be continued by selecting different soils and climate conditions.

Therefore, it is particularly important to perform a study examining how long immobilized microorganisms could survive in mineral fertilizers [41]. Currently, our research results confirm that the bacteria in the pellet remain viable for at least 1000 days, increase the efficiency of mineral fertilizers, stimulate the growth of the plants, and increase microbiological efficiency of the soil and plant productivity (Figure 4D).

Essentially, little change in cell viability and activity were observed. It was concluded that the *Bacillus* sp. MVY-004 microorganism is suitable for the development of a new type of biotechnology—biomineral fertilizers. The use of mineral–chemical fertilizers is increasingly being restricted on the initiative of the European Union because it depends on environmental guidelines [42]. On the other hand, the growth of the biofertilizer market is over 10% per year. Even if at first glance mineral–chemical and biological fertilizers seem like rivals, eventually they can become good allies on the new agrobiotechnology horizon [22,43].

Phosphorus in mineral fertilizers is quickly immobilized and becomes inaccessible to plants. Phosphate solubilizing microorganisms isolated from the agricultural soil improve agricultural plant growth, soil biodiversity, and phosphorus availability converting, insoluble phosphorus forms into soluble forms [44]. Phosphate solubilizing microorganisms are known to be abundant in the rhizo-spheric soils of various plants, but their presence varied considerably according to plant species [8]. Phosphate solubilization potential has been attributed to the microorganisms ability to reduce the pH of the surroundings, or by releasing organic acids [45]. Phosphate solubilizing bacteria belong to genera such as *Bacillus* spp., *Pseudomonas* spp., *Agrobacterium* spp., *Enterobacter* spp., *Rhizobium* spp., and *Burkholderia* spp. [46]. Our solubilization experiments confirmed the earlier finding that the phosphate solubilizing microorganism formed a clear halo zone on PVK agar medium and showed the maximum phosphate solubilization activity, as indicated by the transparent zone formation on PVK agar plates.

Phosphate solubilizers required carbon, organic, nitrogen sources, and mineral salts for energy, both for the synthesis of new cell material and the oxidation of carbon compounds [47]. It is well known that an increasing number of microorganisms are associated with the plant rhizosphere, especially due to its carbon concentration. The growth of the rhizosphere microorganism cells is also influenced by temperature and pH value [48]. The basic function of a fermenter is to provide an environment suitable for the controlled growth of a pure culture. The fermentation process is a great means of obtaining maximum productivity and maintain the most appropriate conditions for growth [48,49].

In addition to phosphorus solubilization activity, the phosphate solubilizing microorganism was reported to secrete phytohormones which might have an influence on plants’ root growth [50]. The root development and plant growth were highly correlated with the higher availability of soluble phosphorus and plant hormone production. They promote the growth of plants by the production of various phytohormones such as indole acetic acid, cyto-kinins, gibberellins, abscisic acid, ethylene, and jasmonic acid [51].

Nowadays, biomineral fertilizer has been increasingly introduced to poor soil to examine its effects on soil and plants [52]. Furthermore, this is expected to be a great alternative in the agricultural sector in order to phase out chemical pesticides and replace them with biological fertilizers. The *Bacillus* genus is preferred for bio-formulations’ preparation because of their long shelf life and their ability to form environmentally resistant spores, which can survive in very extreme conditions [53]. The *Bacillus* genus also possesses several other required characteristics, which include their tendency to replicate at a faster rate, their ability to colonize the roots rapidly, and their competitive colonization potential [54].

In this study, the use of mineral fertilizers with integrated *Bacillus* sp. MVY-004 strain spores showed important results. Biomineral fertilizers increased the amount of soluble phosphorus in loamy soil and minimized mineral fertilizers, the cost of inputs and environmental pollution [20]. The application of *Bacillus* sp. MVY-004 as a biofertilizer could be an alternative option to reduce considerable amounts of mineral fertilizers, increase soil biological activity and agricultural plant production, protect plants from diseases and pathogens, and solve ecological and environmental problems. In the future, experiments and analyses should be performed to test other soil microorganisms or microbial consortiums with mineral/chemical fertilizers. *Bacillus* sp. MVY-004 demonstrated strong application potential and probable utility for future agricultural and biotechnological application.

## 4. Conclusions

In this study, the effective phosphate solubilizing isolate *Bacillus* sp. MVY-004 demonstrated its potential as biomineral fertilizer. This is a very important technology that enhances agroecological practices, more especially phosphorus solubilization, and approved the ability to produce various organic acids and phytohormones as plant growth regulators. Furthermore, efforts should be made to elucidate the mechanism involved in the association between *Bacillus* sp. MVY-004 and different agricultural soil with low phosphorus availability. Future experiments need to be conducted to estimate its growth-promoting performance for different plants. The optimization of the processes of selection, storage of PSM, microorganism cells’ fermentation, and their interactions in the rhizosphere are steps in mastering the development of efficient microbial inoculants with high phosphorus solubilization capacity.

## Figures and Tables

**Table 1:** Calculation of phosphate solubilization index in the PVK agar medium after 2, 5, 7, 10, and 14 days.

Days of Incubation	Colony Diameter (cm)	Solubilization Zone Diameter (cm)	Phosphate Solubilization Index (PSI)
2	0.4 ± 0.08	0.6 ± 0.05	2.50
5	0.8 ± 0.08	1.2 ± 0.04	2.50
7	0.9 ± 0.12	1.5 ± 0.05	2.67
10	1.0 ± 0.12	1.7 ± 0.08	2.70
14	1.2 ± 0.16	2.2 ± 0.08	2.83

**Table 2:** Organic acid detection in *Bacillus* sp. MVY-004 bacterial supernatant using LC-TOF/MS.

Concentration, µg mL^−1^ (Average from Triplicates ± SD)						
Citric Acid *	Succinic Acid **	2-ketogluconic Acid *	Gluconic Acid*	Malic Acid **	Lactic Acid ***	Oxalic Acid ***
13.5 ± 0.6	257.0 ± 3.0	68.0 ± 2.0	287.0 ± 13.0	232.0 ± 7.0	419.0 ± 3.0	34.0 ± 2.0

* concentration calculated using citric acid calibration curve; ** concentration calculated using succinic acid calibration curve; *** concentration calculated using oxalic acid calibration curve.

**Table 3:** Plant hormones detection in *Bacillus* sp. MVY-004 bacterial supernatant using LC-TOF/MS.

Concentration, µg mL^−1^ (Average from Triplicates ± SD)		
Indole-3-acetic Acid *	Jasmonic Acid **	Gibberellic Acid ***
1.370 ± 0.020	0.173 ± 0.009	0.800 ± 0.060

* concentration calculated using indole-3-acetic acid calibration curve; ** concentration calculated using jasmonic acid calibration curve; *** concentration calculated using gibberellic acid calibration curve.

## Data Availability

The genome sequence of *Bacillus* sp. MVY-004 was deposited in NCBI GenBank under the accession number KY882273.
